# Supplementary material for: Characterization of Extracellular Vesicle Cargo in Sjögren’s Syndrome through a SWATH-MS Proteomics Approach
Source: Int J Mol Sci. 2021 May 4;22(9):4864. doi: 10.3390/ijms22094864 (PMC8124455; doi:10.3390/ijms22094864)
Supplement: Supplementary file 1 [file ijms-22-04864-s001.zip › supplementary files final/Figure-S1.pdf]

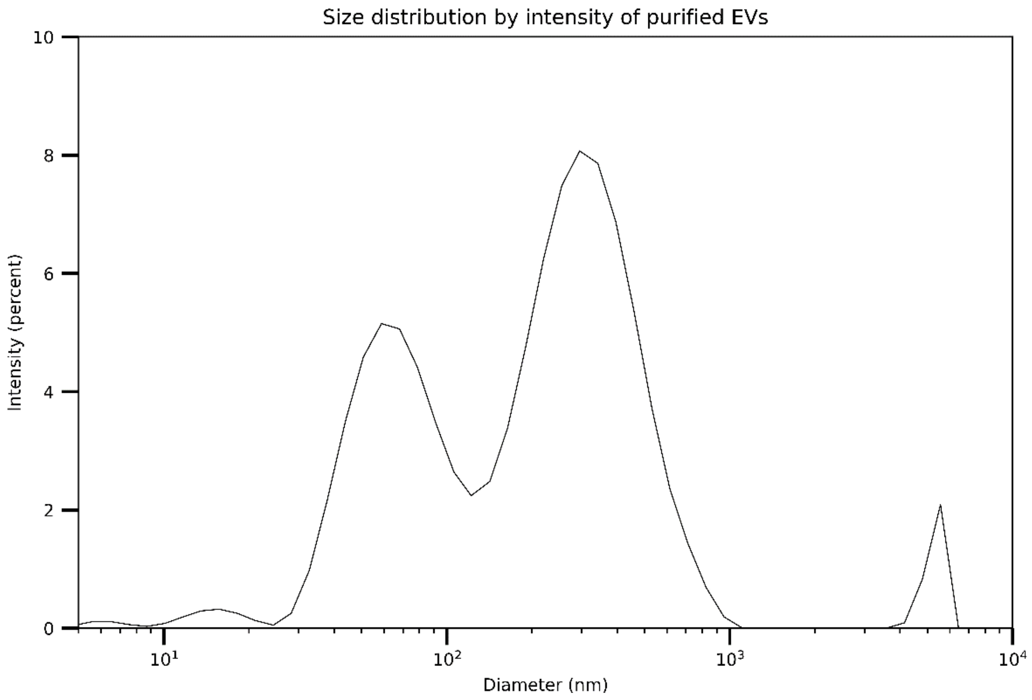

Diameter (nm)	*Relative abundance (%)*
69 ± 24	*61.7*
330 ± 150	*33.7*
5307 ± 395	*2.9*

Figure S1: size distribution and relative percentage abundance (determined based on intensity distribution) of saliva-enriched samples used for the analysis. Reported size distribution is the average of three experiments. Errors are calculated based on the PDI of each vesicle population.
